# A case of irreversible bradycardia after rituximab therapy for diffuse large B-cell lymphoma

**DOI:** 10.1186/s40959-020-00077-5

**Published:** 2020-10-13

**Authors:** Nway Le Ko Ko, Nareg Minaskeian, Hicham Z. El Masry

**Affiliations:** Department of Cardiovascular Medicine, Mayo Clinic Arizona, Phoenix, AZ USA

**Keywords:** Rituximab, High grade AV block, Complete AV block, Bradyarrythmia, Diffuse large B cell lymphoma

## Abstract

This is a case of a middle-aged woman with underlying cardiac conduction system with episodes of AV Wenckebach, who subsequently developed significant AV conduction system abnormalities after receiving one standard dose of Rituximab infusion for diffuse large B-cell lymphoma. Rituximab, being a monoclonal antibody against CD-20 antigen, is effective in treatment of B-cell lymphoma but may also cause bradyarrythmias likely due to the calcium ion channel property of CD-20 antigen.

## Introduction

Rituximab is a monoclonal antibody directed against the CD-20 antigen. The chimeric antibody has been used in the treatment of B-cell lymphoma and binds to the surface antigen activating complement-mediated cell toxicity. Cardiac complications related to Rituximab have been reported including cardiomyopathy and myocardial infarction. Rituximab-induced cardiac arrhythmias have been rarely reported.

We report a patient with sinus dysfunction and paroxysmal high-grade AV block after a single dose of Rituximab used for treatment of diffuse large B cell lymphoma.

## Case

Our patient is a 56-year-old lady with no known cardiovascular condition other than controlled hypertension who presented to our hospital with an insidious onset of bilateral lower extremity lymphedema, and an obstructive uropathy with secondary acute kidney injury. Her initial work up was revealing of a large pelvic mass and extensive lymphadenopathy for which she underwent lymph node, pelvic mass and bone marrow biopsies. A confirmed diagnosis of high-grade lymphoma, most likely a follicular lymphoma transformed into diffuse large B cell lymphoma (DLBCL), required her to be started on R-ECPOCH therapy (Rituximab, Etoposide, Prednisone, Vincristine, Cyclophosphamide and Doxorubicin). Patient reported an excellent pre-morbid functional status, and was a regular volleyball player and a walker prior to the last few months. She received the first dose of Rituximab 375 mg/m^2^ over 36 h along with Methylprednisolone and Doxorubicin as part of her combination chemotherapy. Patient received a total of 800 mg of Rituximab over 36 h.

**Fig. 1:**
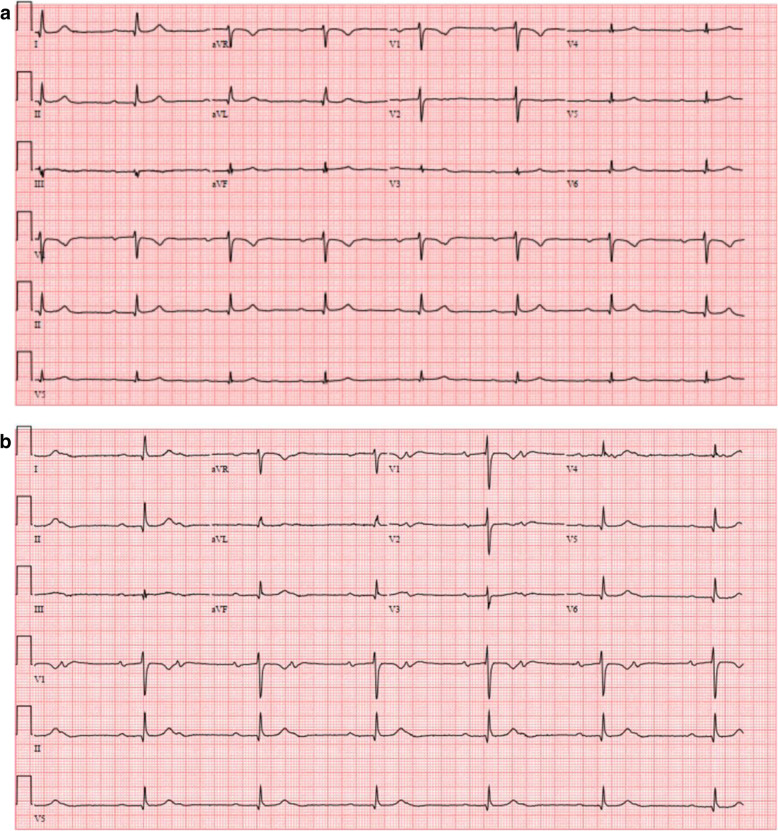
Showing the patient’s serial electrocardiograms: **a** baseline tracing demonstrating sinus rhythm with first degree AV block, **b** sinus rhythm with 2:1 AV block

**Fig. 2:**
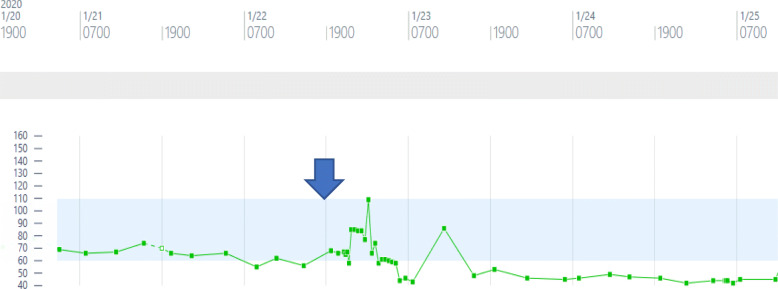
Showing the trend of patient’s heart rate: the arrow signified the time Rituximab therapy was initiated

Upon assessment of the patient, her laboratory parameters revealed chronic microcytic anemia with hemoglobin of 6.9 mg/dl and leukocytosis with white blood cell count of 11 × 10^9^/L with neutrophilia and lymphopenia. Her kidney function and electrolytes were normal. Her echocardiogram was evident for normal biventricular function and global longitudinal strain without any significant valvular anomalies. Initial EKG was apparent for underlying conduction system disease with episodes of AV Wenckebach. In telemetry records, the rhythm had been predominantly in 2:1 AV blocks with additional periods of transient complete AV block with a ventricular rate down to 30s with narrow QRS complexes (Fig. 1). Over the continued observation for 24 h, it was evident that with exercise, her heart rate increased with the rhythm going back into Mobitz type 1 AV block consistent with AV nodal level of conduction block. However, for most of the time, the predominant rhythm was 2:1 AV block with continued occurrence of episodes of high-grade AV block and junctional escape rhythm. Reports of occasional dizziness and an episode of near syncope with worsening bradycardia hence lead to discontinuation of Rituximab therapy as a careful review of her medications did not reveal any negative chronotropic or dromotropic agent that might explain worsening bradycardia (Fig. 2). The calculated Naranjo Adverse Drug Reaction Probability Score of 3 with Rituximab in this case indicated that her AV block was possibly caused by adverse drug effect of Rituximab, as it followed a temporal sequence after Rituximab and it could be explained by the characteristic of her AV block (Supplementary Appendix, Table 1) [1]. Her bradyarrythmia persisted more than 48 h after interruption of her infusion and a pacemaker was implanted at that point. The first time pacemaker interrogation revealed right atrial pacing percentage of 2.3% and right ventricular pacing percentage of 99.3% with the lower rate limit set at 60. The patient received another round of Rituximab in a month. The following pacemaker interrogation revealed right atrial pacing percentage of 6.96% and right ventricular pacing percentage of 99.7% with the lower rate limit set at 60 beats per minute.

## Discussion

Infusion reactions associated with Rituximab therapy have been reported including cardiac arrhythmia such as monomorphic ventricular tachycardia, supraventricular tachycardia, trigeminy, and irregular pulse [2–4]. Moreover, it can also cause bradycardia and AV blocks [2, 5, 6]. Based on our knowledge, this is the second case report of Rituximab causing high grade AV block as there has only been one case report of complete AV block after the fifth dose of Rituximab [5]. In the first reported case, the patient did not have underlying conduction system disease, but our patient did although they both received similar dose of Rituximab per cycle for the indication of DLBCL. In our case, it is likely that the underlying conduction system disease had put our patient into high grade AV block just after the first dose of Rituximab. The half-life elimination of Rituximab is proportional to dosage. After an initial dose of 375 mg/m^2^, the average half-life of Rituximab is 3.2 days (range, 1.3–6.4 days) [7]. For our patient, the AV block onset within 2 days of Rituximab infusion, and her conduction system failed to recover after more than 48 h of monitoring. There have been several explanations of the mechanism of Rituximab affecting the cardiac conduction system. Per Poterucha et.al., it was hypothesized that CD20 antigen may function as a calcium-ion channel. Rituximab can induce the death of CD20+ cells in many ways such as direct cytotoxicity mediated by antibody dependent cells and complement cascade as well as by indirect effects like apoptosis, structural changes and sensitization of malignant cells to chemotherapy [8]. Therefore, it is likely that Rituximab affects cardiac conduction system by inhibiting the calcium-ion-channel properties of the CD20 antigen on the cardiac myocytes. This mechanism appears to be the most plausible pathogenesis of our patient’s bradycardia: infusion initiation was associated with sinus bradycardia and worsening AV block which appears to be at the AV nodal level (improved conduction with exercise and narrow junctional escape with transient high grade AV block). A calcium channel blocking effect at both the sinus and atrioventricular node levels is a unifying explanation of those observations. Other hypotheses include elevated transforming growth factor-B promoting growth of reticulin fiber in cardiac myocytes which impairs contractility and conduction [9] as well as release of cytokines, such as interleukin-6 and tumor necrosis factor-alpha [5]. These cytokines can also mediate ventricular dysfunction, acute coronary syndrome, and myocarditis in addition to tachy-arrhythmias or brady-arrhythmias [10].

## Conclusion

With this case report, we intend to highlight the effect of Rituximab on cardiac conduction due to calcium channel property of CD20 antigen. FDA has cautiously provided recommendations to discontinue infusions for serious or life-threatening cardiac arrhythmias and perform cardiac monitoring during and after each infusion of Rituximab for patients who develop clinically significant arrhythmias, or who have a history of arrhythmia, angina or conduction system disease like our patient [10, 11]. We would also recommend telemetry monitoring for patients who demonstrate any baseline evidence of sinus node dysfunction or conduction abnormalities predisposing them to symptomatic bradycardia with Rituximab therapy and its calcium channel blocking property.

## Data Availability

Not applicable.

## Appendix

**Table 1:** Naranjo Adverse Drug Reaction Probability Scale

Question	Yes	No	Do not know	Score
Are there previous conclusive reports on this reaction?	+ 1	0	0	+ 1
Did the adverse event appear after the suspected drug was administered?	+ 2	− 1	0	+ 2
Did the adverse event improve when the drug was discontinued or a specific antagonist was administered?	+ 1	0	0	0
Did the adverse event reappear when the drug was readministered?	+ 2	−1	0	0
Are there alternative causes that could on their own have caused the reaction?	−1	+ 2	0	0
Did the reaction reappear when a placebo was given?	−1	+ 1	0	0
Was the drug detected in blood or other fluids in concentrations known to be toxic?	+ 1	0	0	0
Was the reaction more severe when the dose was increased or less severe when the dose was decreased?	+ 1	0	0	0
Did the patient have a similar reaction to the same or similar drugs in any previous exposure?	+ 1	0	0	0
Was the adverse event confirmed by any objective evidence?	+ 1	0	0	0
Total score				3
